# Supplementary material for: A whole-animal phenotypic drug screen identifies suppressors of atherogenic lipoproteins
Source: eLife. 2026 Aug 27;14:RP105314. doi: 10.7554/eLife.105314 (PMC13521566; doi:10.7554/eLife.105314)
Supplement: Supplementary file 2 [file elife-105314-code1.zip › JHDL_primary_result/Bismuth (III)┬áoxychloride/Bismuth (III)┬áoxychloride_JHDL_26_038_018_2019_11_08_10_43.pdf]

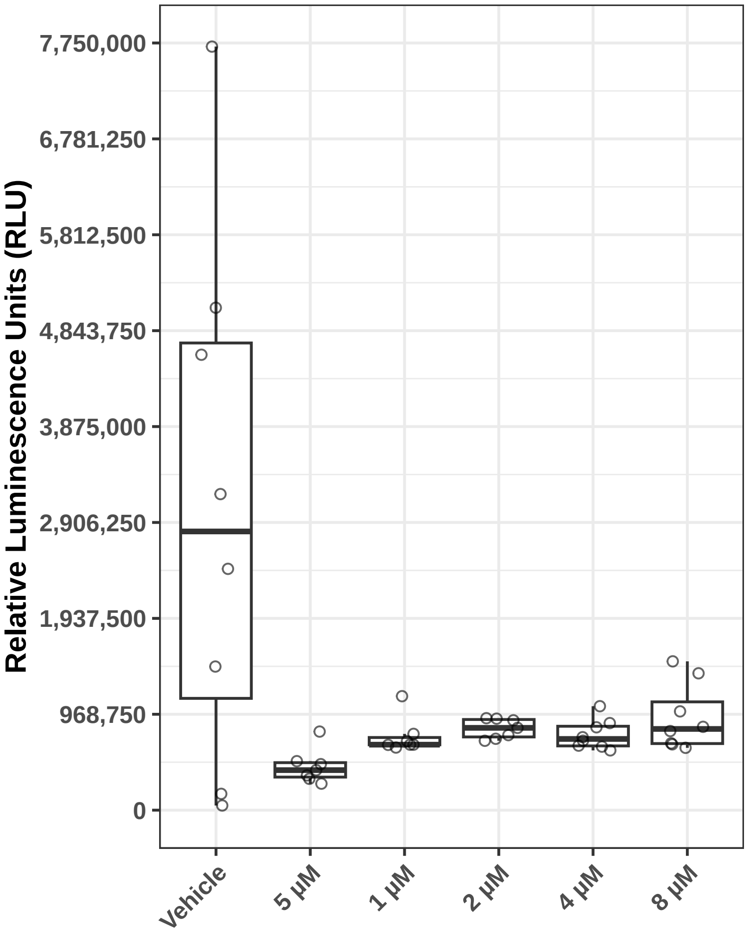
Relative Luminescence Units (RLU)
7,750,000
6,781,250
5,812,500
4,843,750
3,875,000
2,906,250
1,937,500
968,750
0
Vehicle
5 µM
1 µM
2 µM
4 µM
8 µM
